# Efficacy of Mobile Telemedicine System for Digital Subtraction Angiography of Moyamoya Disease Compared with Picture Archiving and Communication System

**DOI:** 10.31662/jmaj.2023-0129

**Published:** 2024-02-09

**Authors:** Toshiya Osanai, Haruto Uchino, Masaki Ito, Miki Fujimura

**Affiliations:** 1 Department of Neurosurgery, Hokkaido University Hospital, Sapporo, Japan

**Keywords:** telemedicine, information and communications technology, moyamoya disease, digital subtract angiography

## Introduction

With the recent advances in telecommunications technology, telemedicine is rapidly developing. The efficacy of conventional telemedicine for stroke has been demonstrated in many studies, and recent guidelines support its use, particularly in areas with poor access to stroke specialists ^(1),(2),(3)^. Mobile systems that use smartphones and other portable devices have been developed and are becoming increasingly convenient ^(4)^. Although telemedicine devices have been developed for stroke treatment, the main imaging modalities are computed tomography (CT) and magnetic resonance imaging (MRI); the diagnostic accuracy of other imaging modalities in telemedicine has not yet been verified. Conversely, digital subtraction angiography (DSA) remains the gold standard for diagnosing cerebrovascular diseases. Moyamoya disease is common in East Asia and causes hemorrhagic and ischemic strokes; the gold standard for the diagnosis of this disease is angiography ^(5)^. Angiographic findings are diverse and require skillful reading. However, there are fewer experts than stroke specialists, possibly due to the rarity of the disease. Thus, teleconsultation may be effective; however, whether the angiographic findings of moyamoya disease can be evaluated using the telestroke system has not yet been confirmed.

This study aimed to verify the diagnostic accuracy of the angiographic findings of moyamoya disease when diagnosed using a mobile telemedicine system. We retrospectively examined the angiographic findings of patients with moyamoya disease who underwent angiography at our hospital and compared them with the picture archiving and communication system (PACS) findings.

## Methods

The study included 15 participants (30 hemispheres) diagnosed with moyamoya disease aged 18 years or older who underwent diagnostic angiography before receiving surgical treatment. Written consent was obtained from all participants for participation in this study.

Our hospital introduced the JOIN (Allm, Tokyo, Japan) system as a remote diagnosis system consisting of a virtual server that can export digital imaging and communications in medicine (DICOM) images from PACS. It is free and available as a mobile application from the Apple Store, Google Play, etc. but requires permission from the hospital administrator for use.

The main functions of the JOIN application used at our institution are chat and viewing DICOM images. DICOM images are encrypted and highly secured. The application runs on a smartphone, and the images can be zoomed in and out with a finger pinch.

In the evaluation, one author (T.O) randomly uploaded images from the list of cases to JOIN, and readers 1 (HU) and 2 (MI) read and recorded the predetermined findings on their smartphones blindly. Then, Readers 1 and 2 read the images on PACS as the gold standard in the same blind mode.

The DSA endpoints were as follows: Suzuki stage ^(5)^, collateral channels (including the lenticulostriate artery (LSA), thalamic artery, and choroidal artery), the posterior cerebral artery (PCA) stenosis, and transdural anastomosis ^(6)^. Intermodality and interrater agreement were calculated for the Suzuki stage, whereas sensitivity, specificity, positive predictive value (PPV), negative predictive value (NPV), and accuracy were calculated for the remaining endpoints. Prism (GraphPad Software, San Diego, CA, USA) was used to conduct statistical analysis.

## Results

**Figure 1.**
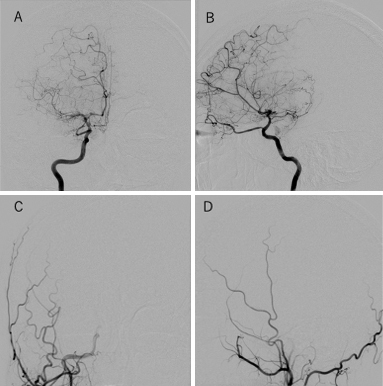
Reader 1 evaluated one hemisphere by JOIN as differing by more than two stages from that of PACS. (A) Frontal view of the right internal carotid artery, (B) lateral view of the right internal carotid artery, (C) frontal view of the right external carotid artery, and (D) lateral view of the left external carotid artery.

Both raters evaluated all cases using their smartphones via a mobile application. Reader 1 assigned two hemispheres to Suzuki Stage 1, eight to Stage 2, seven to Stage 3, seven to Stage 4, four to Stage 5, and two to Stage 6 on the mobile application. Contrarily, in PACS, Reader 1 assigned one hemisphere to Suzuki Stage 1, six to Stage 2, 11 to Stage 3, six to Stage 4, four to Stage 5, and two to Stage 6. Using JOIN, Reader 1 overestimated three and underestimated five hemispheres when compared with PACS for the Suzuki stage. For one hemisphere, Reader 1 disagreed with PACS by more than two stages (Figure 1). The kappa value between JOIN and PACS was 0.79.

Reader 2 assigned two hemispheres to Suzuki Stage 1, six to Stage 2, three to Stage 3, 10 to Stage 4, three to Stage 5, and five to Stage 6 on the mobile application. Contrarily, in PACS, Reader 2 assigned 2 hemispheres to Suzuki Stage 1, 7 to Stage 2, 2 to Stage 3, 12 to Stage 4, 6 to Stage 5, and 1 to Stage 6. Using JOIN, Reader 2 overestimated eight and underestimated three hemispheres when compared with PACS for Suzuki stage. Reader 2 never disagreed with PACS by more than two stages. The kappa value between JOIN and PACS was 0.77 (Table 1 and 2). The interrater agreement in JOIN was 0.747.

**Table 1.** Distribution of Suzuki Classification Number by Reader 1 with PACS and JOIN.

		PACS					
		1	2	3	4	5	6
JOIN	1	1	1				
	2		5	2	1		
	3			6	1		
	4			3	4		
	5					4	
	6						2

PACS: Picture Archiving and Communication System

**Table 2.** Distribution of Suzuki Classification Number by Reader 2 with PACS and JOIN.

		PACS					
		1	2	3	4	5	6
JOIN	1	1	1			
	2	1	5			
	3		1	1	2	
	4			1	9		
	5				1	2
	6					4	1

PACS: Picture Archiving and Communication System

Regarding LSA collaterals, Reader 1 had a sensitivity, specificity, PPV, NPV, and accuracy of 75%, 88.5%, 50%, 95.8%, and 86.7% whereas Reader 2 had 0%, 96.3%, 0%, 89.7%, and 86.7%, respectively.

For thalamic collateral, Reader 1 had a sensitivity, specificity, PPV, NPV, and accuracy of 100%, 86.2%, 20%, 100%, and 86.7% whereas Reader 2 had 100%, 100%, 100%, 100%, and 100%, respectively.

For choroidal collateral, Reader 1 had a sensitivity, specificity, PPV, NPV, and accuracy of 66.7%, 83.3%, 50%, 90.9%, and 80% whereas Reader 2 had 80%, 100%, 100%, 96.2%, and 96.7%, respectively.

For PCA stenosis, Reader 1 had a sensitivity, specificity, PPV, NPV, and accuracy of 75%, 96.2%, 75%, 96.2%, and 93.3% whereas Reader 2 had 50%, 88.5%, 40%, 92%, and 83.3%, respectively.

For transdural anastomosis, Reader 1 had a sensitivity, specificity, PPV, NPV, and accuracy of 94.4%, 91.7%, 94.4%, 91.7%, and 93.3% whereas Reader 2 had 87.5%, 71.4%, 77.8%, 83.3%, and 80%, respectively (Table 3).

**Table 3.** Sensitivity, Specificity, Positive Predictive Value, Negative Predictive Value, and Accuracy of Digital Subtraction Angiography (DSA) Endpoints for Each Reader.

		Sensitivity (%)	Specificity (%)	PPV（%）	NPV (%)	Accuracy (%)
LSA collateral	Reader1	75	88.5	50	95.8	86.7
	Reader2	0	96.3	0	89.7	86.7
Thalamic collateral	Reader1	100	86.2	20	100	86.7
	Reader2	100	100	100	100	100
Choroidal Collateral	Reader1	66.7	83.3	50	90.9	80
	Reader2	80	100	100	96.2	96.7
PCA stenosis	Reader1	75	96.2	75	96.2	93.3
	Reader2	50	88.5	40	92	83.3
Transdural anastomosis	Reader1	94.4	91.7	94.4	91.7	93.3
	Reader2	87.5	71.4	77.8	83.3	80

LSA: lenticulo striatum artery; PCA: posterior cerebral artery; PPV: positive predictive value; NPV: negative predictive value

## Discussion

This study shows that even with DSA, which has more complex findings than MRI and CT, the mobile application exhibited a substantial level of diagnostic accuracy compared with the regular PACS. More detailed findings than those previously reported for common ischemic strokes, such as collateral vessels, arterial stenosis, and transdural anastomosis in moyamoya disease, could be confirmed on JOIN.

Mobile applications have the advantage of being portable, enabling physicians to obtain DSA results without worrying about their location or the need to visit a hospital. JOIN is expected to reduce the burden on physicians and has been considered an effective tool for “reforming the practice of doctors,” which will begin in Japan in April 2024. Reform in the practice of physicians will require fewer physicians, and information and communications technology (ICT) is expected to be used to solve this problem; however, how ICT will be exactly used is still being discussed. This research is expected to lead to applications in a wider range of modalities and other diseases, not just brain diseases.

In previous studies, telemedicine has exhibited sensitivity, specificity, and accuracy close to 100% ^(4),(7)^; however, the results of our study are not as good as those of previous studies. The diagnostic modalities employed in previous studies were CT and MRI, although the DSA results in this study are subject to different interpretations, and there is variation in interreader agreement even on PACS. DSA in moyamoya disease requires more skill in angiographic reading, such as the Suzuki stage and fine collateral vessels, than in other diseases. Therefore, the telemedicine results in this study were considered satisfactory. Furthermore, the indications for bypass surgery in moyamoya disease must be determined based on angiographic findings and symptoms, and teleconsultation using JOIN is expected to allow for the consideration of optimal indications for treatment.

In conclusion, the preoperative DSA findings of moyamoya disease can be sufficiently evaluated using a mobile device. Further validation using other modalities and diseases will enable a wider range of telemedicine applications.

## Article Information

### Conflicts of Interest

None

### Sources of Funding

TO, HU, and MF belong to the endowed course sponsored by Allm Inc. MF is one of the editors of the JMA Journal.

### Author Contributions

All authors contributed to the content and writing of the manuscript. TO wrote the first draft of the manuscript. HU and MI contributed to the collection of data. MF contributed to the supervision of the research activity. All authors contributed to the scientific content of the manuscript, critically reviewed it, and approved the final version. All authors made the final decision to submit the paper for publication

### Approval by Institutional Review Board (IRB)

14-053 at Hokkaido University Hospital

### Disclaimer

Miki Fujimura is one of the Editors of JMA Journal and on the journal’s Editorial Staff. He was not involved in the editorial evaluation or decision to accept this article for publication at all.
